# Assessing Performance on Digital Clock Drawing Test in Aged Patients With Cerebral Small Vessel Disease

**DOI:** 10.3389/fneur.2019.01259

**Published:** 2019-11-26

**Authors:** Hóngyi Zhào, Wei Wei, Ellen Yi-Luen Do, Yonghua Huang

**Affiliations:** ^1^Department of Neurology, Seventh Medical Center of PLA General Hospital, Beijing, China; ^2^Department of Neurology, No. 984 Hospital of the People's Liberation Army, Beijing, China; ^3^ATLAS Institute, University of Colorado Boulder, Boulder, CO, United States

**Keywords:** aging, assessment, digital technology, executive function, small vessel disease

## Abstract

The term small vessel disease (SVD) encompasses all the pathological processes that affect the small vessels of the brain, including small arteries and arterioles but also capillaries and small veins, which can result in multi-domain cognitive deficits. The digital clock drawing test (dCDT) has been proved to be a more useful assessment tool for cognitive disorders compared to traditional clock drawing test DT (tCDT) in many neuropsychological diseases. This study aimed to check whether this tool worked well in capturing some specific aspects of cognitive performance in aged patients with SVD. A total of 20 aged patients with high-burden SVD (severe-SVD), 10 aged patients with low burden SVD (low-SVD), and 10 age-matched (healthy) individuals were grouped according to Fazekas' score. The dCDT and a series of neuropsychological assessments were performed to evaluate the cognitive function of participants. severe-SVD patients showed higher air-time percentage and lower mean handwriting/drawing pressure on surface during drawing compared with low-SVD and healthy subjects. The linear regression analysis adjusted for age, gender and education showed that the air-time percentage during drawing correlated with the choice reaction test (CRT) and the digit symbol substitution test (DSST), and the mean handwriting/drawing pressure on surface showed a limited correlation with DSST. The data indicated that some early manifestations of cognitive deficits in aged patients with SVD could be detected using the dCDT with a brand-new perspective different from the tCDT.

## Introduction

The term “cerebral small vessel disease (SVD)” has gained attentions in cerebrovascular practice because it is becoming one of the most common causes of vascular cognitive impairment and vascular dementia (1). The relevant deficits of SVD mainly involve cognitive flexibility, attention, and processing speed, with episodic memory, naming, and orientation relatively spared (2–4).

The clock drawing test (CDT) is one of the most popularly used tests by neuropsychologists because it provides an economical and comprehensive evaluation of multiple cognitive domains. The traditional CDT (tCDT) is brief, acceptable to patients, easy to score, relatively independent of educational/cultural/language confounders, psychometrically robust, and broad in its coverage of cognitive domains. The tCDT demands such as drawing and handwriting are complex human activities that entail an intricate blend of cognitive, kinesthetic, and perceptual-motor components (5). Poor performance on the tCDT could imply the cognitive dysfunctions though recent researches focusing on SVD patients did not come to unanimous findings (6). This might be caused by the lack of dynamic and kinematic variables in the tCDT. As we know, the tCDT were used to detect dementia, or differentiating different types of dementia (7) through scoring system evaluating the elements of content and structure in the final product as a whole, rather than detecting the process step by step. Besides cognitive domains, motor ability of is also commonly affected in SVD patients (especially during fulfillment of motor-cognitive incorporated dual tasks), which could be expressed as “Motoric-cognitive risk syndrome” (8). The digital clock drawing test (dCDT) conserves the score system of tCDT. Moreover, capturing and monitoring data through computerization provides additional dimensions such as air-time during drawing, pressure on surface, and so forth (9). Thus, the advantages of the dCDT allow for a closer inspection of successful clock drawing performance including the efficient use of time and graph-motor output to facilitate performance. Nowadays, the dCDT has been proved to be acceptable to detect cognitive impairments in many neuropsychiatric disorders (10, 11).

The present study was to find the dCDT performance regarding to the dynamic and kinematic results, as well as relevant cognitive declines in the elderly with different levels of SVD burden.

## Methods

### Participants

From June 1, 2018 to December 31, 2018, 20 aged patients with high-burden SVD (severe-SVD), 10 aged patients with low burden SVD (low-SVD), and 10 age-matched (healthy) were recruited and screened by 3.0 T MRI brain and grouped according to the Fazekas' scale (12). Severity of white matter lesions was graded as: no lesions (grade 0), punctate lesions (grade 1), early confluent lesions (grade 2), and confluent lesions (grade 3). Exclusion criteria is subjects with major strokes or cerebral bleedings, other causes of leukoencephalopathy (e.g., immune, demyelination, genetic); major psychiatric diseases; use of psychotropic medications, multisystem diseases, and MRI contraindications; other non-vascular dementia. All participants in this study were right-handed and had normal visual acuity and comprehensive capacity. General information, including name, sex, age, and years of education, was collected from participants.

### Neuropsychological Assessment

All participants completed a series of neuropsychological assessment, including MMSE (reflecting global cognitive level), choice reaction test (CRT) (reflecting attention and concentration), digit symbol substitution test (DSST) (reflecting executive function and processing speed), category verbal fluency test (cVFT) (reflecting executive function), and trail making test (TMT) -B (reflecting cognitive flexibility).

**Figure 1:**
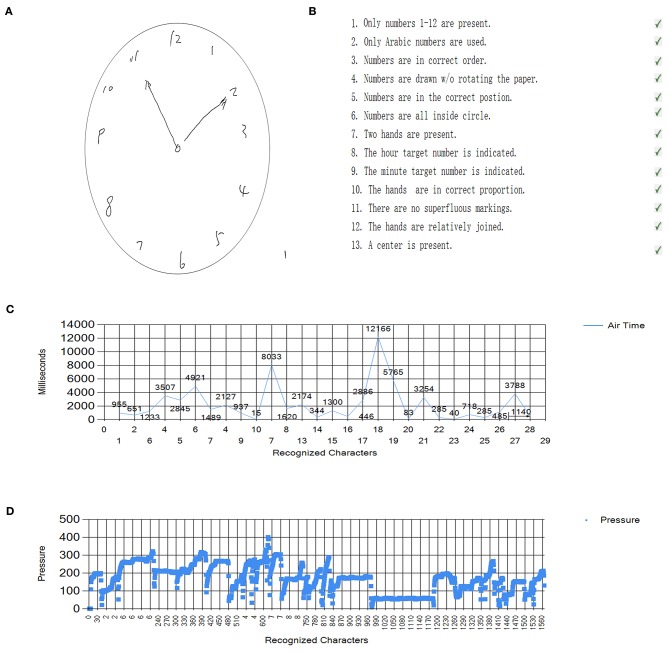
Example of dCDT results of participants. **(A)** The participants were asked to “draw the face of a clock with all of the numbers and set the hands to 10 after 11.” **(B)** The drawing score system was assessed according to the 13-point criteria of Freedman. **(C)** The air-time was captured, and percentage was calculated by dividing the total time to fulfill clock drawing. **(D)** Mean handwriting/drawing pressure during the dCDT drawing (implemented on the writing surface of computer screen in non-scaled units from 0 to 1,024).

The dCDT software was previously used as a feasible cognitive assessment tool for dementia screening (13). Briefly, a Windows Surface Pro 4 digitizer and a handheld stylus pen were used to assess drawing movements. This software was designed to check drawing elements of the dCDT, including air-time (the time accumulated when pen pressure was 0 during clock drawing) and total time for the dCDT completement in milliseconds, and mean handwriting/drawing pressure during the dCDT drawing (implemented on the writing surface of computer screen in non-scaled units from 0 to 1,024). The participants were asked to “draw the face of a clock with all of the numbers and set the hands to 10 after 11.” The drawing score system was assessed according to the 13-point criteria (14). Examples of dCDT measurements, including tCDT pictures, 13-point scoring system, air-time percentage [(air-time/total time) × 100%] calculation and mean handwriting/drawing pressure calculation are shown in Figure 1.

### MRI Measurements

The 3.0 T-MRI scan (Discovery MR750; GE Healthcare, Waukesha, WI, USA) of the brain showed white matter lesions compatible with a degree of SVD. The brain imaging protocol (slice thickness, 5 mm; interslice thickness, 1.5 mm) employed the following parameters: T1 fluid-attenuated inversion recovery images (TR, 1,750 ms; TE, 23 ms; TI, 780 ms; FOV, 24 cm) and T2-weighted images (TR, 7,498 ms; TE, 105 ms; FOV, 24 cm).

**Figure 2:**
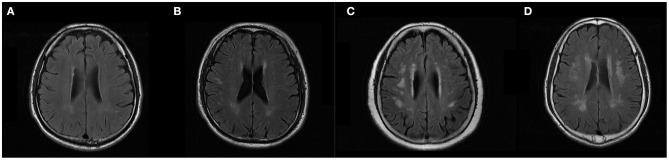
Example of brain T2 Flair MRI of subjects. According to the Fazekas' score (12), severity of white matter lesions was graded as: no lesions (grade 0), punctate lesions (grade 1), early confluent lesions (grade 2), and confluent lesions (grade 3). **(A)** was rated as 0 and grouped as healthy individuals. **(B)** was rated as 1 and grouped as low-SVD. **(C,D)** were rated as 2 and 3, respectively. They were grouped as severe-SVD.

The degree of SVD burden level was evaluated according to the Fazekas' scale (12). Examples of MRI brain images of each group were shown in Figure 2.

### Statistical Analysis

The differences between the groups demographic data were analyzed by one-way analysis of variance (ANOVA). The differences between the groups on MMSE, CRT, DSST, cVFT, TMT-B performance as well as dCDT data (including the tCDT score, total time of CDT fulfillment, air-time percentage, and mean handwriting/drawing pressure on surface) were analyzed by analysis of covariance (ANCOVA), with the age and education serving as the covariate. Follow-up comparisons of estimated marginal means using least squares difference (LSD) was performed as needed. A linear regression analysis adjusted for age, gender and education was used to find the association between dCDT data and neuropsychological assessment performance, and the regression coefficients was presented as standardized β values. The data were expressed as the mean ± standard deviation. A *P* value <0.05 was considered statistically significant. All statistical analyses were carried out using the SPSS, 22.0 statistical software package (IBM Corp., Armonk, New York, USA).

## Results

Severe-SVD, low-SVD, and healthy group individuals did not show significant difference in age, gender, and education. After adjusting for age and education, the score in MMSE was lower in severe-SVD compared with low-SVD and healthy individuals, but the difference did not reach statistical significance (26.25 ± 1.45 vs. 27.60 ± 1.17 vs. 26.80 ± 2.04, *P* = 0.813). Meanwhile, each group of subjects did not differ significantly in cVFT performance (14.45 ± 3.24 vs. 16.60 ± 4.37 vs. 14.70 ± 1.82, *P* = 0.686). On the contrary, CRT (572.25 ± 125.53 ms vs. 489.30 ± 99.06 ms vs. 472.40 ± 96.14 ms, *P* = 0.030), DSST (18.75 ± 7.33 vs. 27.70 ± 9.44 vs. 27.20 ± 8.45, *P* = 0.036), and TMT-B (89.30 ± 28.98 s vs. 74.00 ± 20.47 s vs. 65.70 ± 18.97 s, *P* = 0.047) differed statistically between group subjects. Details were shown in Table 1.

**Table 1:** Clinical and demographic characteristics and dCDT data of the participants.

	**Healthy individuals (*N* = 10)**	**Low-SVD (*N* = 10)**	**Severe-SVD (*N* = 20)**	***P* value**
Men, %	60%	70%	55%	0.747
Age, years	71.30 (4.47)	71.30 (5.05)	71.85 (5.34)	0.943
Education, years	7.20 (1.31)	8.50 (2.87)	9.05 (3.30)	0.249
MMSE, score	26.25 (1.60)	27.20 (1.64)	26.25 (1.45)	0.813
CRT, milliseconds	472.40 (96.14)	489.30 (99.60)	572.25 (125.54)	0.030^*^^#^
DSST	27.20 (8.45)	27.70 (9.44)	18.75 (7.33)	0.036^*^
cFVT	14.70 (1.82)	16.60 (4.37)	14.45 (3.24)	0.686
TMT-B, seconds	65.70 (18.97)	74.00 (20.47)	89.30 (28.98)	0.047^*^
tCDT, score	10.40 (0.84)	9.90 (1.20)	9.80 (1.39)	0.440
Total time, seconds	53.90 (16.40)	65.30 (16.83)	71.55 (43.75)	0.401
Air-time percentage, %	61.90 (7.94)	62.00 (8.40)	68.90 (8.34)	0.039^*^^#^
Pressure	485.20 (162.65)	428.60 (139.60)	368.45 (109.48)	0.037^*^

*Mean (Standard Deviation)*.

* *P < 0.05 severe-SVD relative to low-SVD*.

# *P < 0.05 severe-SVD relative to healthy individuals. Comparison of MMSE, CRT, DSST, cVFT, TMT-B performance as well as dCDT data (including tCDT score, Total time, Air-time percentage, and Pressure) were adjusting for age and education as covariate*.

The command condition tCDT score did not differ statistically between groups, although severe-SVD group patients scored lower than low-SVD group patients healthy individuals (9.80 ± 1.39 vs. 9.90 ± 1.20 vs. 10.40 ± 0.84, *P* = 0.440) with adjustment of age and education. The same phenomenon was found in total time of CDT fulfillment (71.55 ± 43.75 s vs. 65.30 ± 16.83 s vs. 53.90 ± 16.40 s, *P* = 0.401), as shown in Table 1, severe-SVD group patients required more total time to complete drawing test than low-SVD group patients and healthy individuals, but the difference did not reach significance.

Analyzing the digitized variables during clock drawing, the air-time percentage was found to be significantly different between severe-SVD and other two groups, higher air-time percentage was found in severe-SVD group patients compared with low-SVD group patients and healthy individuals (68.90 ± 8.34% vs. 62.00 ± 8.40% vs. 61.90 ± 7.94%, *P* = 0.039) (Shown in Table 1). The data in Table 1 also revealed that the mean handwriting/drawing pressure on surface was statistically lower in severe-SVD group patients compared with low-SVD group patients and healthy individuals during the CDT (368.45 ± 108.48 vs. 428.60 ± 139.60 vs. 485.20 ± 162.65, *P* = 0.037).

Further, the association between cognitive task score and digitized variables during clock drawing was explored using linear regression analysis adjusted for age, gender, and education. The air-time percentage positively correlated with CRT (*P* = 0.017, standardized β = 0.374), and negatively correlated with DSST (*P* = 0.005, standardized β = −0.446). However, mean handwriting/drawing pressure on surface did not correlate with performance in neuropsychological assessments performed in the present study. Details were shown in Table 2.

**Table 2:** Association between the dCDT data and neuropsychological assessment performance.

	**Air-time percentage, %**		**Pressure**	
	**Standardized β value**	***P* value**	**Standardized β value**	***P* value**
**MMSE, score**				
Model 1	−0.016	0.920	0.262	0.103
Model 2	−0.038	0.822	0.149	0.380
**CRT, milliseconds**				
Model 1	0.381	0.015^*^	0.081	0.618
Model 2	0.374	0.017^*^	0.083	0.614
**DSST**				
Model 1	−0.482	0.002^#^	−0.254	0.114
Model 2	−0.446	0.005^#^	−0.010	0.955
**cFVT**				
Model 1	−0.036	0.826	0.228	0.072
Model 2	−0.013	0.935	0.270	0.096
**TMT-B, seconds**				
Model 1	0.228	0.072	−0.254	0.114
Model 2	0.226	0.196	−0.227	0.202

*Data are standardized β values, except for P value. Model 1 represents the relation between the dCDT data and neuropsychological assessment performance without adjustment. Model 2 represents the relation between the dCDT data and neuropsychological assessment performance adjusted for age and education*.

* *P < 0.05*.

# *P < 0.01*.

## Discussion

In the present study, the performance of patients with severe-SVD on the dCDT was found poorer compared with patients with low-SVD and healthy individuals, reflected in air-time percentage and mean handwriting/drawing pressure on surface, rather than clock drawing score and total time for completing the drawing. Also, patients with severe-SVD showed several cognitive deficits, including attention/concentration, processing speed, executive function, and cognitive flexibility compared with other group of participants. Furthermore, for aged individuals, the dCDT air-time percentage was associated with CRT and DSST performance. Whereas, similar trends were not found in mean handwriting/drawing pressure on surface.

The air-time percentage during the drawing task was considered as representing unobservable cognitive activities such as motor planning and programming (15). In old patients with depression, Cohen et al. (10) found that the “Think time percentage” of the dCDT (similar to “air-time percentage” in our study) negatively correlated with attention/information processing. Another study launched by Müller et al. (16) demonstrated that the “time-in-air” of the dCDT (similar to “air-time percentage” in our study) was significantly longer in patients with amnestic mild cognitive impairment (MCI) compared with healthy controls, even in patients amnestic MCI having the normal conventional CDT score. The present study involved old patients with SVD and age-matched healthy controls. It also found that the air-time percentage was significantly higher in patients with severe-SVD relative to patients with low-SVD and healthy individuals.

Another interesting finding of the current study was the distinct pressure on surface between group participants during clock drawing. Werner et al. (17) have reported that the mean pressure during handwriting was found to be lower in more cognitively deteriorated elderly compared with healthy controls. The pressure data in their study were collected while completing a series of Hebrew alphabet handwriting tasks. Moreover, in the study launched by Garre-Olmo et al. (18), aged participants were asked to complete more complicated tasks including sentences handwriting, 3D house drawing, and clock drawing. The results suggested that “on surface pressure” during writing and drawing might be a useful and objective complement to distinguish patients with Alzheimer's Disease and MCI from healthy elderly people. The findings of the present study revealed that lower pressure on surface during clock drawing in patients with severe-SVD relative to patients with low-SVD and controls. Despite adopting different tasks, a decrease in pressure during handwriting and drawing was found in many neurological diseases (11).

Two factors which did not reach significance for patients with severe-SVD and other two group of participants: CDT score and total dCDT fulfilling time. Quite a few studies showed that the cognitive decline in patients with SVD was characterized by executive dysfunction, but it was difficult to measure using normal screening tools like conventional CDT, because of ceiling effects (19). This could explain the negative results on these two factors in the present study. However, the dCDT could be a proper assessment tool, because it used air-time percentage and pressure during drawing, rather than the final product, to detect the cognitive deficits in patients with SVD.

Apart from air-time percentage and pressure findings between groups, the linear regression analysis demonstrated that dCDT performance was associated with some neuropsychological dimensions in aged participants. The air-time percentage correlated with CRT and DSST. CRT and DSST were known to reflect attention/concentration and processing speed, which could be a possible explanation for the air-time percentage elevation in patients with SVD during dCDT drawing. Although the pressure on surface during dCDT was found to be lower in patients with severe-SVD, no correlation was found between pressure and neuropsychological assessments. This might be because of the lack of a sufficient number of participants or neuropsychological assessments. Further studies regarding in the pressure on surface during a dCDT are needed to elucidate the underlying mechanism.

Several limitations of this study warrant consideration. First, the sample size was small. Second, the CDT, no matter traditional or digital, could be measured through a command and copy conditions, which demanded different cognitive load (10). This study chose a command instead of the copy condition CDT, considering the ceiling effect of the executive decline in patients with SVD. Third, some aspects, such as memory and orientation ability, were not chosen in the present study because patients with SVD were characterized not to show deficits in these domains (2–4). In future study, we would collect more participants and encompass more tests to overcome these limitations.

In conclusion, aged participants with a high SVD burden were found to show dCDT deficits than those with low SVD burden. The dCDT deficits lied in air-time percentage and mean handwriting/drawing pressure on surface, rather than in clock drawing score and total time for completing the drawing. The air-time percentage of dCDT was associated with CRT and DSST performance which were representations of attention/concentration and executive function and processing speed, respectively. In summary, these data indicated that the dCDT could be used to find several aspects of cognitive decline in aged patients with SVD with a brand new perspective different from the tCDT.

## Data Availability Statement

All datasets generated for this study are included in the article.

## Ethics Statement

The studies involving human participants were reviewed and approved by the ethics committee of the Seventh Medical Center of PLA General Hospital. And all subjects gave written informed consent in accordance with the Declaration of Helsinki. The patients/participants provided their written informed consent to participate in this study.

## Author Contributions

HZ and WW were responsible for data collection and analysis. ED was responsible for software maintenance. YH was responsible for manuscript preparation.

### Conflict of Interest

The authors declare that the research was conducted in the absence of any commercial or financial relationships that could be construed as a potential conflict of interest.
